# Biological ageing and the risk of decreased handgrip strength among community-dwelling older adult Indians: a cross-sectional study

**DOI:** 10.1186/s12877-023-04498-6

**Published:** 2023-11-28

**Authors:** Vishal Vennu

**Affiliations:** Department of Rehabilitation Sciences, College of Applied Medical Sciences, King Saud University, Riyadh, Saudi Arabia

**Keywords:** Biological ageing, Older adults, Hand dynamometer, Handgrip strength

## Abstract

**Background:**

Evidence from the literature demonstrates that the risk of decreased handgrip strength is associated with various health issues, particularly in older persons. To make judgments regarding their general health condition that are well-informed for longevity, it is crucial to assess the risk level of decreased handgrip strength among community-dwelling older adult Indians. However, no study has examined the relationship between biological aging and the risk of decreased handgrip strength in Indian men and women aged 60 and older. The goal of the current study was to fill this gap in the literature.

**Methods:**

In this cross-sectional study, we included 31,464 (15,098 men and 16,366 women) community-dwelling older adult Indians aged 60 years and older using data from the Longitudinal Aging Study in India (LASI). The LASI is the world’s most extensive and India’s first multidisciplinary, internationally harmonized, longitudinal aging study. It has enrolled 72,250 individuals aged 45 and above across all 28 states and 8 union territories of India. Secondary analysis of biological ageing was performed by stratifying for age groups (60–64, 65–69, 70–74, 75–79, 80–84, and 85 + years) for both genders. The dominant right and nondominant left handgrip strength was assessed using the portable Smedley’s Hand Dynamometer. All individuals had a dominant right hand. The adjusted logistic regression analysis assessed the association between biological ageing and the risk of decreased handgrip strength for both genders.

**Results:**

Compared to those between the ages of 60–64, those at age 65 and those aged 85 and above had 1-fold and 12-fold odds of decreasing handgrip strength, respectively. Men 85 years or older had a 12-fold higher chance than women in the same age group of having decreased handgrip strength.

**Conclusions:**

The results indicate that community-dwelling older adult Indians aged 65 years and older are significantly associated with a higher risk of decreased handgrip strength, especially among older men. The results of this study can help assess and implement handgrip strength measurement in medicine for older Indians as part of regular admission assessment, particularly for older men.

## Introduction

Given the ageing population around the world [1], handgrip strength declines with age [2]. Thus, it eventually starts to have an impact on daily activities that are made more or less difficult based on the physical performance of simple tasks like opening jars, carrying groceries, and turning doorknobs [3]. It is also a trustworthy predictor of many age-related health issues. For instance, recent research [4, 5] indicated that decreased grip strength was independently linked to a higher risk of cardiovascular mortality, cancer, and dementia from all causes. According to another recent study, men who have diabetes are more likely to have poorer handgrip strength levels, which likely come before the triglyceride glucose index [6]. Additionally, sarcopenia and mild cognitive impairment had a significant association in this sample of older adults living in low- and middle-income countries, particularly in older adult Indians [7].

The proportion of people in India who are 60 years or older is predicted to rise dramatically by 12.5% in 2030 and 19.4% in 2050, in accordance with the evolving patterns of the human ageing [8, 9]. The capacity of these growing populations to carry out survival duties independently and without aid provides insight into how well they are currently doing [10]. Additionally, it helps develop rules that offer better management and outcome evaluation. However, the ability of older people to perform tasks is evaluated using a variety of objective measurements [11–13]. Any health professional can use handgrip strength to anticipate this capacity in any setting [14]. One of the most trustworthy measures of hand strength is handgrip strength, which is straightforward to administer [15]. Many global studies, including longitudinal studies, have found that handgrip strength is an excellent predictor of ability, well-being, and all mortality events in the elderly [16–18]. However, few studies [13, 19, 20] from India attempted to explain the older Indian age group, gender, ethnicity, occupation, culture, or handicap, even though they did demonstrate a sizable difference.

According to a recent study, handgrip strength is associated with disability and self-rated health’s moderating and mediating function [13]. Another recent study found that, compared to those with the lowest handgrip strength, those with higher handgrip strength had lower probabilities of experiencing depressive symptoms by 30% and 47%, respectively, for people 50 to 64 and those 65 years and older [21]. Recent literature reviews and meta-analyses have also shown a connection between lower muscle strength and worsened depressive symptoms in older populations [22]. A new longitudinal study of 115,601 older adults from 24 countries found a dose-response relationship between handgrip strength and the risk of depression [23]. A secondary analysis of the National Health and Nutrition Examination Survey from 2011 to 2014 showed a substantial inverse relationship between handgrip strength and depression in community-dwelling, non-institutionalized people in the United States aged ≥ 60 years [24]. Furthermore, people with low handgrip strength had a higher mortality risk than normal [25, 26]. Also, the findings of a recent study indicate that inadequate handgrip strength may serve as a partial mediator of the effect of anemia on health-related quality of life [27]. According to recent studies [28–30], decreased handgrip strength has been linked to both men’s and women’s measures of neurocognitive brain health. It may also interfere with the metabolism of many anticholinergic medications that are commonly used by older people (e.g., furosemide and selective serotonin reuptake inhibitors), and they may interact with one another and raise the risk of mortality.

Therefore, to ensure that decisions about the older population’s general health status are well-informed for long life. It is vital to evaluate the risk in handgrip strength among community-dwelling older adult Indians by the biological ageing [14, 31]. Thus, this study aimed to investigate the association between biological ageing and the risk of decreased handgrip strength in Indian men and women aged 60 years and older. The assumption is that as a person ages, their likelihood of having a risk in handgrip strength increases, especially among older men in India. This assumption was made as a result of a recent study that found that older Indian men were more likely to have weak handgrips than women due to financial empowerment, which is mostly handled by men in India [32].

## Materials and methods

This study is based on data from the Longitudinal Aging Study in India (LASI). LASI is the world’s most extensive and India’s first multidisciplinary, internationally harmonized, longitudinal aging study. It has enrolled 72,250 individuals aged 45 and above across all states and union territories of India. A multistage stratified area probability cluster sampling design was used for the LASI survey. Previous studies have provided a detailed survey design and data collection methodology [13, 33, 34]. The Indian Council of Medical Research (ICMR) provided the appropriate guidelines and ethics approval for the LASI survey. Before being enrolled in the study, every household and age-eligible person consented.

The participants comprised 15,098 men and 16,366 women, stratified into six age groups (60–64, 65–69, 70–74, 75–79, 80–84, and 85 + years). Individuals younger than 60 (n = 37,029) or with missing handgrip data (n = 3,757) were excluded from the present analysis. Handgrip strength was determined using data from 31,464 adults aged 60 years and above using Smedley’s hand dynamometer by adjusting the respondent’s dominant right and nondominant left hands [16]. The right forearm was placed at the upper arm’s elbow, and the upper arm was kept close to the torso. The subject was instructed to press the dynamometer three times with each hand as firmly as possible for a brief period. As for the grip strength, the highest of the six values was picked. All individuals had a dominant right hand. The average of two consecutive trials in the dominant and nondominant hands was used to obtain the final handgrip strength score in kilograms (kg).

Individuals’ socio-demographic variables, such as gender, educational status, religion, social group, marital status, and place of residence, were collected using a structured questionnaire. Body Mass Index (BMI) was calculated by dividing weight in kg by height in meters (m) squared. The continuously attended school was likewise divided into yes and no categories. The level of education was broken down into primary school, secondary/matriculation, diploma and certificate holders, graduates, post-graduates, and professional degree holders. The terms “Hindu, Muslim, Christian, Sikh, Buddhist/neo-Buddhist, Jain, None, or Others” were used to categorize various religions. Three levels of marital status were categorized as married, widowed/divorced/separated, and never married.

### Statistical analysis

All analyses were performed using SAS, version 9.4 (SAS Institute, Inc., Cary, NC). The Shapiro-Wilk test was used to assess the normality distribution of data. The mean and standard deviation (SD) for continuous variables and the count (%) for categorical variables were used to represent participant characteristics. The significant difference between the genders was determined using a Chi-square test for frequencies and an independent student t-test for mean values. Men and women in six age groups (60–64, 65–69, 70–74, 75–79, 80–84, and 85 + years) had their left and dominant right hands measured for the average mean, SD, and 95% confidence intervals (CI). To ascertain whether there were any differences between the groups, the analyses were performed using the ANOVA test for each age group. The present study’s average values of dominant right handgrip strength of older men and women matched with norms of other countries by age groups (60–64, 65–69, 70–74, and 75 + years) were also presented.

The logistic regression analysis evaluated the association between all six age groups and the risk of decreased handgrip strength in both genders. The analysis was adjusted for age (continuous), ever-attended school, education, religion, marital status, place of residency, and BMI. Given this study’s sample size of 31,464, the study was sufficiently powered (≥ 80%) to detect this association. The reference group for this study comprised people between the ages of 60 and 64. The odds ratios (OR) and 95% CI were used to present the results. The variance inflation factor (VIF) was used to evaluate multicollinearity. A p-value of less than 0.05 was regarded as significant for each analysis.

## Results

**Fig. 1:**
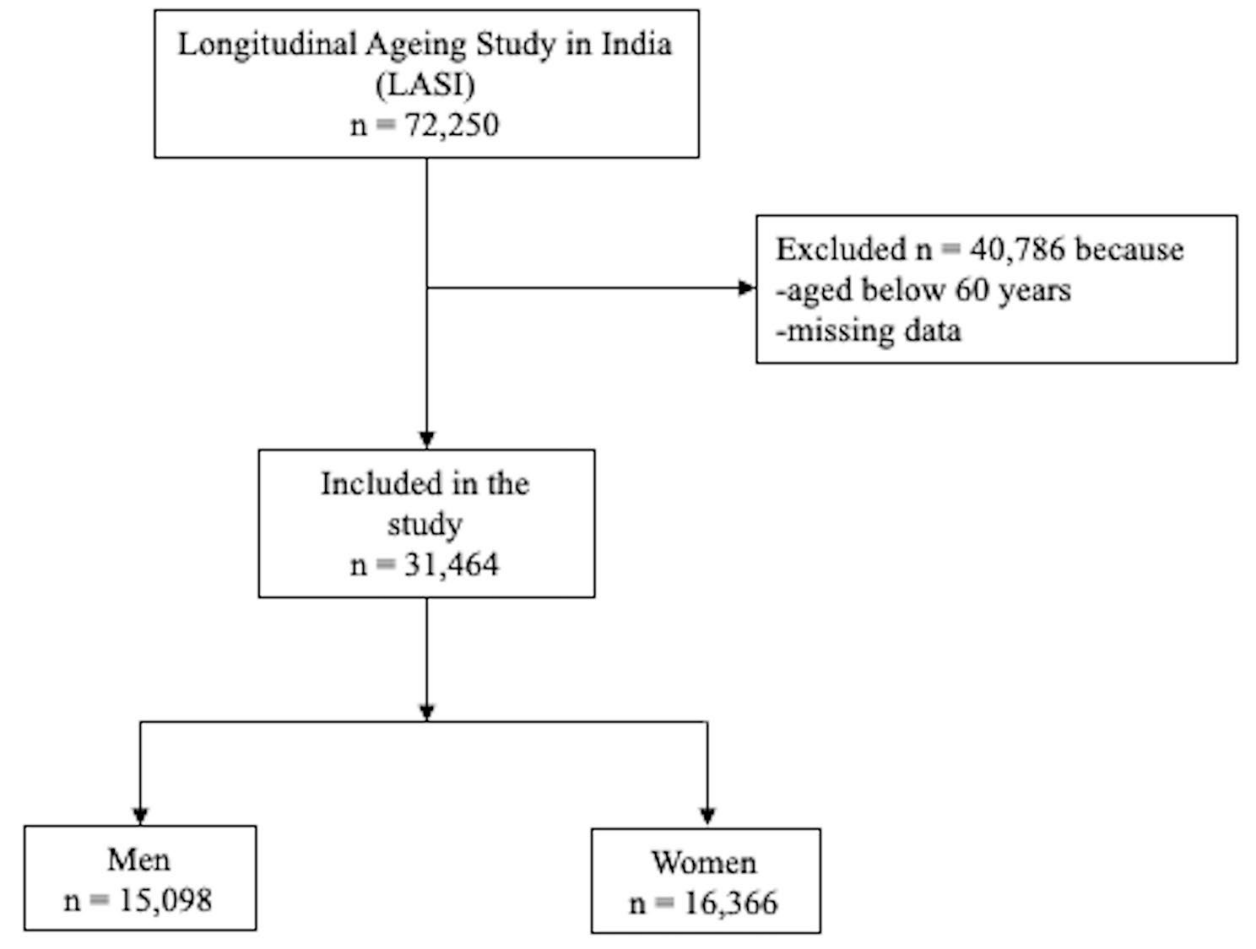
The flow of the study sample

Out of a total of 72,250 participants, 31,464 (46.5%) individual data (15,098 men and 16,366 women) were used in the analysis after 40,786 (56.5%) were excluded. The data were excluded from the study due to their age and lack of data (Fig. 1). The average age of the participants was 68.8 years, with significant differences between genders. Men and women aged 60–64 years were outnumbered (32.2% and 35.3% of the total, respectively). All six age groups significantly (*p < .001*) differ by gender. Most participants (53.7%) did not attend school, particularly women (66%), which was statistically significant (*p < .001*). Most participants had the highest level of education up to the 7th standard (25.9%), particularly women, who significantly had the 4th standard (32.2%). Most participants were married (63.3%), but most women were widowed, divorced, or separated (53.1%). Most maximum participants lived in rural areas (65.8%), especially men (66.7%). Women had a lower average BMI (16.7 kg/m^2^) than men (17.4 kg/m^2^). Men had higher handgrip strength on average (20.5 kg on average for the dominant right and nondominant left hands and 24.7 kg for the dominant hand) than women (18.6 kg on average and 22.7 kg for the dominant hand) (Table 1).

**Table 1:** Characteristics of the sample

	Total n = 31,464	Men n = 15,098 (48%)	Women 16,366 (52%)	*p-Value*
**Age in years, mean ± SD** ^¶^	68.8 ± 7.4	69.0 ± 7.3	68.7 ± 7.6	*0.001*
**Age group, n (%)** ^#^				*<0.001*
60–64	7,007 (33.7)	3,331 (32.2)	3,676 (35.3)	
65–69	5,684 (27.4)	2,923 (28.2)	2,761 (26.5)	
70–74	3,514 (16.9)	1,849 (17.9)	1,665 (16)	
75–79	2,070 (10)	1,060 (10.2)	1,010 (9.7)	
80–84	1,042 (5)	543 (5.2)	499 (4.8)	
≥85	1,448 (7)	648 (6.3)	800 (7.7)	
**Ever attended school, n (%)** ^#^				
Yes	14,575 (46.3)	9,619 (67.6)	4,956 (34)	*<0.001*
No	16,889 (53.7)	5,479 (32.4)	11,410 (66)	
**Year of school, mean ± SD** ^¶^	7.2 ± 3.9	7.9 ± 4.1	6.6 ± 3.7	*<0.001*
**Highest level of education, n (%)** ^#^				*<0.001*
Less than primary school (standard 1–4)	3,781 (25.9)	2,184 (22.7)	1,597 (32.2)	
Primary school (standard 4–7)	3,779 (25.9)	2,295 (23.9)	1,484 (29.9)	
Middle school (standard 8–9)	2,238 (15.4)	1,555 (16.2)	683 (13.8)	
Secondary/Matriculation	2,376 (16.3)	1,752 (18.2)	624 (12.6)	
Intermediate/senior secondary	946 (6.5)	708 (7.4)	238 (4.8)	
Diploma and certificate holder	146 (1)	105 (1.1)	41 (0.8)	
Graduates	815 (5.6)	624 (6.5)	192 (3.9)	
Post-graduates	258 (1.8)	206 (2.1)	52 (1)	
Professional Degree	235 (1.6)	190 (2)	45 (0.9)	
**Religion, n (%)** ^#^				*0.861*
Hindu	23,037 (73.2)	11,078 (73.4)	11,959 (73.1)	
Muslim	3,731 (11.9)	1,804 (11.9)	1,927 (11.8)	
Christian	3,150 (10)	1,468 (9.7)	1,682 (10.3)	
Sikh	979 (3.1)	481 (3.2)	498 (3)	
Buddist/neo-Buddhist	209 (0.7)	105 (0.7)	104 (0.6)	
Jain	73 (0.2)	33 (0.2)	40 (0.2)	
None	65 (0.2)	30 (0.2)	35 (0.2)	
Others (Jewish, Parsi, or Zoroastrian)	219 (0.7)	99 (0.7)	120 (0.7)	
**Caste category, n (%)** ^#^				*0.252*
Scheduled caste (SC)	5,140 (16.9)	2,448 (16.7)	2,692 (17.1)	
Scheduled Tribe (ST)	5,173 (17)	2,436 (16.6)	2,737 (17.3)	
Other backward class (OBC)	11,886 (39.1)	5,781 (39.5)	6,105 (38.7)	
None of them	8,218 (27)	3,970 (27.1)	4,248 (26.9)	
**Marital status, n (%)** ^#^				*<0.001*
Married	19,920 (63.3)	12,398 (82.7)	7,522 (46.1)	
Widow/Divorced/Separated	11,073 (35.4)	2,414 (16.1)	8,659 (53.1)	
Never married	301 (1)	178 (1.2)	123 (0.8)	
**Place of residence, n (%)** ^#^				0.002
Rural	20,717 (65.8)	10,073 (66.7)	10,644 (65)	
Urban	10,747 (34.2)	5,025 (33.3)	5,722 (35)	
**Body mass index (kg/m** ^2^ **), mean ± SD** ^¶^	17.0 ± 3.6	17.4 ± 3.4	16.7 ± 3.9	*<0.001*
**Handgrip strength (kg), mean ± SD** ^¶^				*<0.001*
Dominat right hand	20.5 ± 3.5	24.7 ± 3.4	16.3 ± 3.6	
Nondomiant left hand	18.6 ± 3.8	22.7 ± 3.9	14.6 ± 3.7	

^¶^ Significant difference between groups was determined using the independent student t-test

^#^ Significant difference between groups was determined using the Chi-square t-test

**Fig. 2:**
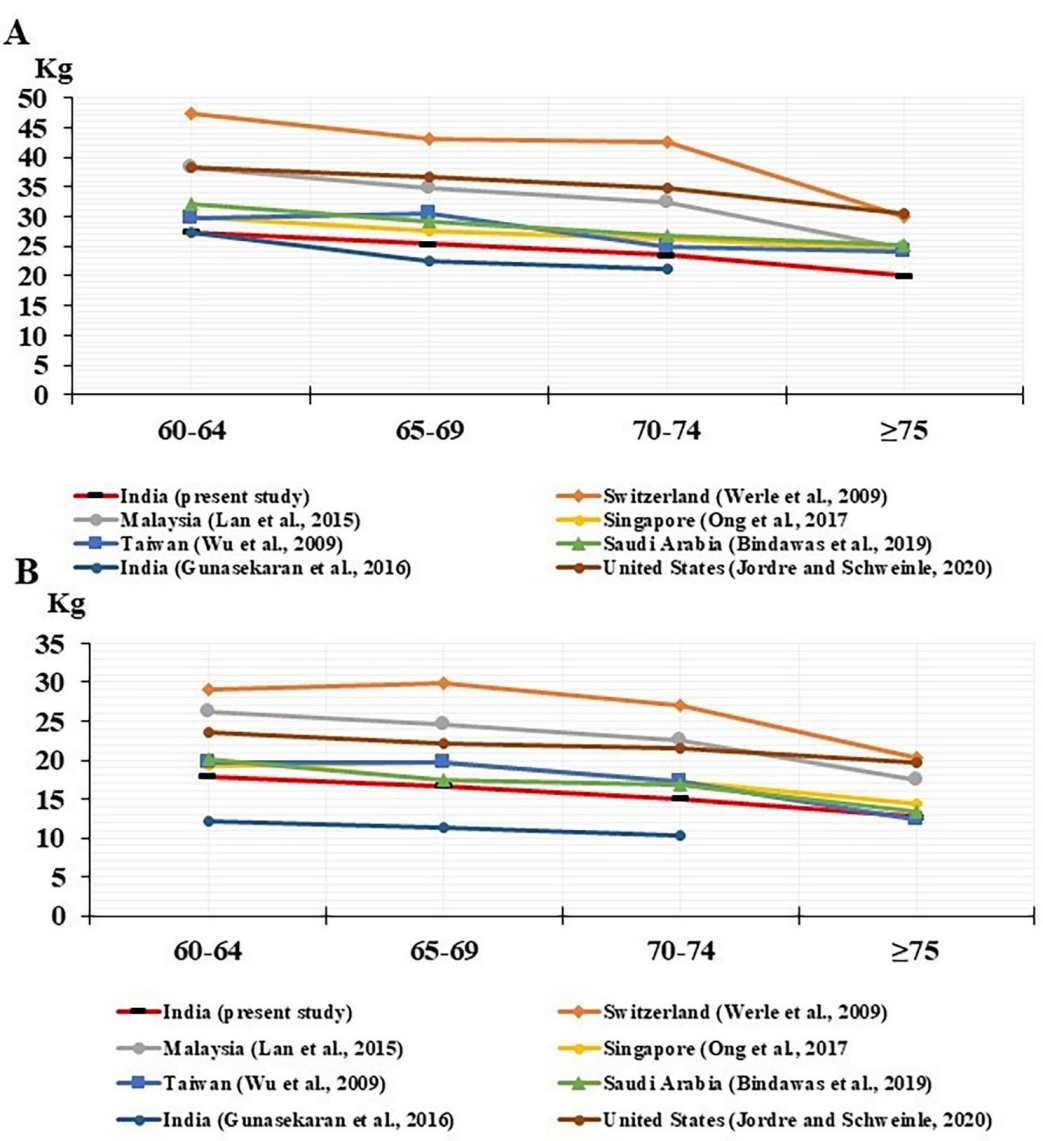
The average handgrip strength of the dominant right hand of older Indian (**A**) men and (**B**) women compared with other countries

The majority of older men (n = 3,331) and women (n = 3,676) were between the ages of 60 and 64, and mean handgrip strength for both dominant and nondominant hands was significantly (*p < .001*) higher in this group (26.3 kg for men and 17.1 kg for women). Raising the age from 60 to 64 years to 85 years significantly (*p < .001*) reduced the mean handgrip strength for both dominant and nondominant hands (16.8 kg for men and 10.8 kg for women) (Table 2). The dominant handgrip strength of older men (32.9 kg) and women (21.9 kg) aged 60–64 years was significantly higher. Each of the five age groups (65–69, 70–74, 75–79, 80–84, and 85 + years) was significantly associated with a greater than 1-to 12-fold odds of handgrip strength as compared to the age group of 60–64 years. Men 85 years or older had a 12-fold higher chance than women in the same age group of having decreased handgrip strength (Fig. 2).

**Table 2:** Descriptive statistics for handgrip strength by gender, hand, and age group

Age group	Men (n = 15,098)			
	**Dominant right hand**		**Nondominant left hand**	
	**Mean ± SD**	**95% CI**	**Mean ± SD**	**95% CI**
60–64	27.4 ± 7.0	27.1—27.6	25.2 ± 6.8	25.0—25.5
65–69	25.4 ± 7.0	25.1—25.6	23.3 ± 6.6	23.0—23.5
70–74	23.5 ± 6.8	23.2—23.8	21.5 ± 6.5	21.1—21.8
75–79	21.9 ± 6.6	21.4—22.3	20.0 ± 6.3	19.6—20.4
80–84	20.4 ± 6.4	19.9—21.0	18.2 ± 6.1	17.7—18.8
≥ 85	17.7 ± 6.2	17.1—18.2	15.9 ± 5.8	15.4—16.4
	**Women (n = 16,366)**			
60–64	17.9 ± 5.0	17.7—18.1	16.2 ± 4.7	16.0—16.3
65–69	16.7 ± 5.0	16.5—16.9	15.0 ± 4.7	14.8—15.1
70–74	15.1 ± 4.8	14.8—15.3	13.4 ± 4.5	13.2—13.7
75–79	13.9 ± 4.6	13.6—14.2	12.5 ± 4.4	12.2—12.7
80–84	12.8 ± 4.5	12.4—13.3	11.2 ± 4.2	10.8—11.6
≥ 85	11.5 ± 4.5	11.1—11.9	10.2 ± 4.0	9.8—10.5

Abbreviations: CI, confidence interval; SD, standard error.

## Discussion

This study investigated the association between biological ageing and the risk of decreased handgrip strength in Indian men and women aged 60 years and older. The key findings revealed that older populations in India, particularly among men, were much more at risk for reduced handgrip strength as they became older, especially those who were 65 years and older. The findings also indicated that handgrip strength decreased with age in older Indian people of both genders and was comparable to norms from Saudi Arabia, Singapore, and Taiwan.

In this study, handgrip strength in both genders was observed to be reduced as age progressed. These observations were broadly similar to the results of other studies with ample justification for separate norms by gender, hand, and age group [35–37]. In a prior study [38], it was found that women’s lower handgrip strength over the age of 55 than in men and decreased with age. Another recent study [39] reported that sarcopenia was found to be more common in males than in women among Indian adults aged 50 and above (37% in men and 17% in women). Another interesting finding of this study was that both the dominant right and nondominant left hands of women aged 60–64 years had weaker grip strength than observed in men of a likewise age group (approximately 9 kg less in both hands). However, the difference between genders seen in the current study was slight compared to other studies that recorded differences of 11 kg [35], 12.5 kg [40], and 15.2 kg [8, 41] in the same age group. A possible reason might be associated with physical determinants, dietary factors, and the overall well-being of the older population.

**Fig. 3:**
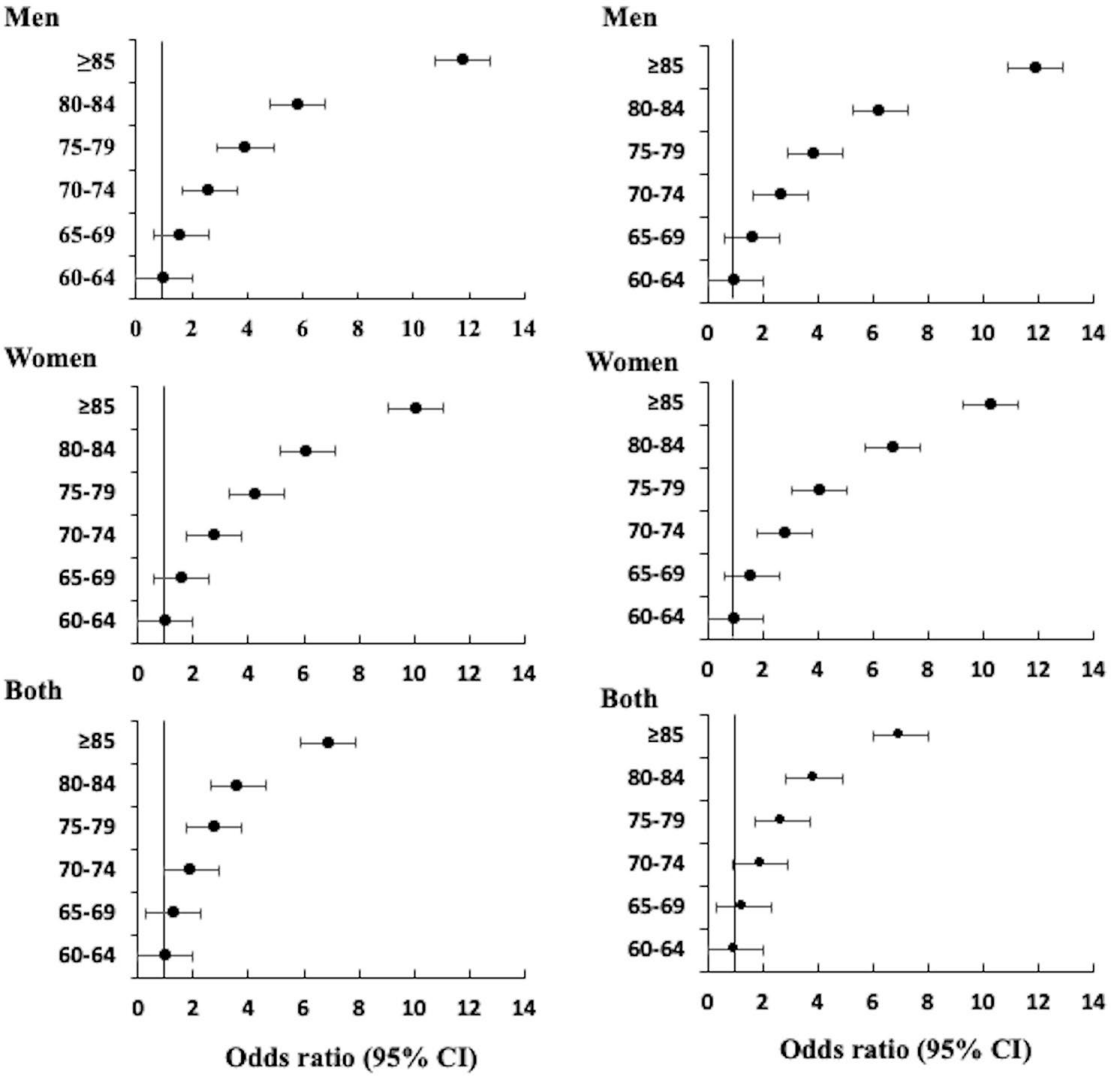
Association between gender age range and handgrip strength risk in community-dwelling older adult Indians. Left said images show the dominant right handgrip strength risk level

This study demonstrated that the mean dominant right handgrip strength among Indian older men was almost identical to the standards of Singapore [42], Taiwan [43], and an earlier Indian study [8]. Also, the average dominant right handgrip strength among Indian older women was similar to those of Saudi Arabia [35] and Taiwan [43]. However, handgrip strength for community-dwelling older adult Indians was lower than the norms of various other countries (Fig. 3) [40, 44, 45]. A recent study [2] indicated that older persons in India, who were 50 years of age and older, had significantly poorer handgrip strength than their counterparts in the other four countries of South Africa, Russia, Ghana, and China. Previous reports from around the world imply that lower handgrip strength is a plausible explanation for these cross-national disparities in grip strength [46–48]. However, research has indicated that variations in race, ethnicity, stature, and body size may influence cross-national variances in handgrip strength [49, 50]. Furthermore, these disparities most likely reflect the variety of individual socioeconomic circumstances, dietary habits, health behaviors, and environmental characteristics that are social determinants of health within a given country [51, 52]. Given this, the results point to the necessity of further research examining the relationship between socioeconomic status and handgrip strength in each country to determine what is normal and what is unique.

The results of this study partially diverge from those of a recent research [13, 20] that was carried out in India utilizing a nationally representative sample. According to the studies, grip strength has a substantial relationship with socioeconomic status, particularly the wealth quintile, but this relationship is narrowed in older persons, particularly among men. Additionally, older persons with insufficient handgrip strength were more likely to experience functional challenges with daily activities, daily living instruments, and low self-rated health. None of these investigations, however, found a connection between biological ageing and the possibility of deteriorating handgrip strength. Additionally, these studies [13, 20, 39] were surveys conducted among people in a few Indian states who were 50 years of age or older.

Findings of this study demonstrate the important role of handgrip strength in biological ageing and monitoring changes in vitality, depression, physical function, and other risk factors for healthy ageing in a developing country like India [2, 53]. Findings also highlight the need to remember that the World Health Organization’s Guidelines on Integrated Care for Older People (ICOPE) support enhancing physical and mental capacity with a thorough strategy catered to each older adult’s unique needs and goals, including multimodal exercise, nutritional interventions, and cognitive stimulation, supported by suitable health and social care systems and service providers [53, 54]. Although lower handgrip strength has been linked to depression [22, 23] and other conditions, such as future fall [55], functional difficulties in activities of daily living [13, 53], disability [56, 57], a higher prevalence of cancer [58], and short-term mortality [59] in old age, greater discussion in the context of biological ageing is necessary to determine the relevance of these findings for public policy. However, there are signs of what can help older persons live better lives, such as policies and initiatives that support better nutrition and target older populations in low-resource sectors. Handgrip strength can be increased with currently available therapies, such as increasing protein intake [60]. To promote healthy ageing, more steps must be taken to lessen the disparity in access to proper nutrition, for instance by focusing on people of lower socioeconomic levels [50].

The primary strength of the present study is the first study that assessed the association between biological ageing and the risk level of decreased handgrip strength for community-dwelling men and women aged 60 years and above, using large data from LASI carried in all 28 states and 8 union territories of India. Earlier studies explored how grip strength has been associated with depression [22, 23] and other health conditions [56, 57]. Also, the present study’s participants differ from the above-referenced other studies, probably owing to variations in age, recruitment, and geographical region [31, 35, 42, 43, 47]. For example, studies [8, 39] generated the level of handgrip strength by recruiting adult participants from a few Indian states [13, 20, 39] or a single-center Geriatric Medicine Clinic [8, 39]. Moreover, handgrip strength was measured with a well-accepted, reliable, and valid tool hand dynamometer for community-dwelling older adults [42]. Other strengths are that this is the first study that reported grip strength by gender, hand, and age groups. In addition, this study had a good representative sample for all six age groups to accommodate handgrip for this community-dwelling older population. However, the present study has a limitation in the absence of palm length, upper arm, participant’s height, and waist circumferences [42] along with the participant’s hand sensations [61]. These factors might have influenced the validity of the study results.

## Conclusion

Biological ageing was significantly linked to a higher risk of decreased handgrip strength in community-dwelling older Indian, particularly among older men. However, handgrip strength in this population is similar to Saudi Arabians, Singaporeans, and Taiwanese normative values. A thorough geriatric assessment, which takes into account handgrip strength, is required to better identify the likelihood that older people will have a bad prognosis. The findings of this study may be useful in determining handgrip strength measurements for older Indians as part of routine admittance assessments. The association with several influential factors in this population must be investigated through prospective studies.

## Data Availability

The study is based on LASI data available for free public access at the International Institute for Population Science: https://www.iipsindia.ac.in/content/LASI-data.
